# A fully contained sample holder capable of electron-yield detection at soft X-ray energies

**DOI:** 10.1107/S1600577524011354

**Published:** 2025-01-01

**Authors:** S. Olivia Gunther, Patrick W. Smith, Jacob A. Branson, Alexander S. Ditter, Stefan G. Minasian, Alpha T. N’Diaye, Bianca Schacherl, David K. Shuh

**Affiliations:** a Lawrence Berkeley National Laboratory Berkeley CA94720 USA; ESRF – The European Synchrotron, France

**Keywords:** soft X-rays, soft X-ray absorption spectroscopy, XAS, X-ray magnetic circular dichroism, XMCD, total electron yield, holders

## Abstract

A holder has been developed that enables electron yield-detected soft X-ray spectroscopy of fully contained samples at low temperature. Crucially, this design uses elements of the sample containment to collect ejected electrons, removing the need to expose samples directly to the vacuum environment of the spectrometer. The design is modular and should be adaptable to a number of different endstation configurations, enabling spectroscopy of air-sensitive, radioactive and vacuum-sensitive (biological) samples.

## Abstract 2

Demonstration of a fully contained sample holder for electron yield-detected soft X-ray spectroscopy.

## 1. Introduction

Soft X-ray absorption spectroscopy (XAS, 100–2000 eV) of fully contained samples remains challenging due to the conflicting requirements of sample protection, X-ray transmission and signal detection. Sample holders must be sufficiently robust to ensure sample integrity while also featuring a thin membrane that transmits the beam with minimal attenuation. Additionally, at soft X-ray energies, the short absorption length of most materials necessitates alternative techniques such as scanning transmission X-ray microscopy (STXM) (Smiles *et al.*, 2020 ▸; Bluhm *et al.*, 2006 ▸). Fluorescence yield (FY) measurements have also been used previously for encapsulated samples (Shuh *et al.*, 2003 ▸), but not all endstations incorporate appropriate fluorescence detectors and FY spectra are often distorted relative to XAS (Kurian *et al.*, 2012 ▸). Flourescent decay of core holes that are excited by absorption in the soft X-ray range is much less likely then Auger decay. Hence, fluorescence detectors need to cover a large solid angle and are particularly uncommon in techniques where space in the vicinity of the sample is constrained, as in magnetic spectroscopies such as X-ray magnetic circular dichroism (XMCD).

At soft X-ray energies, the dominant mode of core-hole quenching is via the Auger effect (Krause, 1979 ▸; Meddouh *et al.*, 2023 ▸), and so total electron yield (TEY) is often used to measure X-ray absorption. This comes with the additional advantages of high detection efficiency and does not impose the stringent requirement of a uniform and X-ray transparent sample that is needed for the direct detection of X-ray transmission. This detection scheme is typically performed with the samples uncontained in the vacuum environment of the endstation, which can be difficult to achieve without degradation of air-sensitive samples and is incompatible with samples that cannot be exposed to the vacuum chamber (*e.g.* radioactive materials). Here we combine the needs of sample encapsulation, protection and detection by demonstrating a holder that enables soft X-ray TEY detection for fully contained samples.

Two strategies that have been employed for electron yield detection of air-sensitive samples include construction of a transfer chamber that can interface with the endstation (Schneider *et al.*, 2020 ▸; Weinhardt *et al.*, 2021 ▸) and preparation of the sample *in situ* by deposition (Kummer *et al.*, 2016 ▸). Specialized equipment is needed for both of these approaches, and the latter strategy has the additional limitation that only volatile samples (or those that can be synthesized *in situ*) may be used. Notably, the sample is not contained throughout the process and measurement using either of these approaches, rendering them unsuitable for samples that must additionally be isolated from the experimental chamber. Thus, there is a clear need for a sample holder that can enable TEY detection while also providing a protecting barrier between the sample and both air and vacuum.

## 2. Results and discussion

In the past, a number of alternative TEY detection schemes have been employed that inspired our approach. At hard and tender X-ray energies (above 5 keV), TEY has been recorded in a grazing incidence holder using a positively charged electrode placed near the sample (Poswal *et al.*, 2020 ▸). Liquid holders capable of soft X-ray spectroelectrochemistry have also been reported, where the sample is contained by a gold-coated Si_3_N_4_ window that acts in tandem as a working electrode, TEY detector and X-ray transparent window (Velasco-Velez *et al.*, 2014 ▸; Wu *et al.*, 2018 ▸; Kao *et al.*, 2020 ▸; Van Spronsen *et al.*, 2021 ▸). Combining these two approaches, a holder was designed whereby samples rest on a conductive support. It is then hermetically sealed under an inert atmosphere to a silicon frame that suspends an Si_3_N_4_ membrane above the sample. A gasket of aluminized Kapton (polyimide) provides for electrical insulation between the base with the sample as a cathode and the window as an anode. With a potential difference applied between the base and window, the ejected electrons from the sample can be detected as a drain current. Though the holder is sealed under an inert atmosphere, species resulting from electron–gas collision will remain charged. An applied voltage of 10 V–50 V ensures that emitted electrons and these potential ionization products drift to the electrodes and contribute to the TEY current. For molecular samples and other insulators, preparation of sufficiently thin films avoids charging effects that can otherwise distort the spectra (Karweik & Winograd, 1976 ▸).

**Figure 1:**
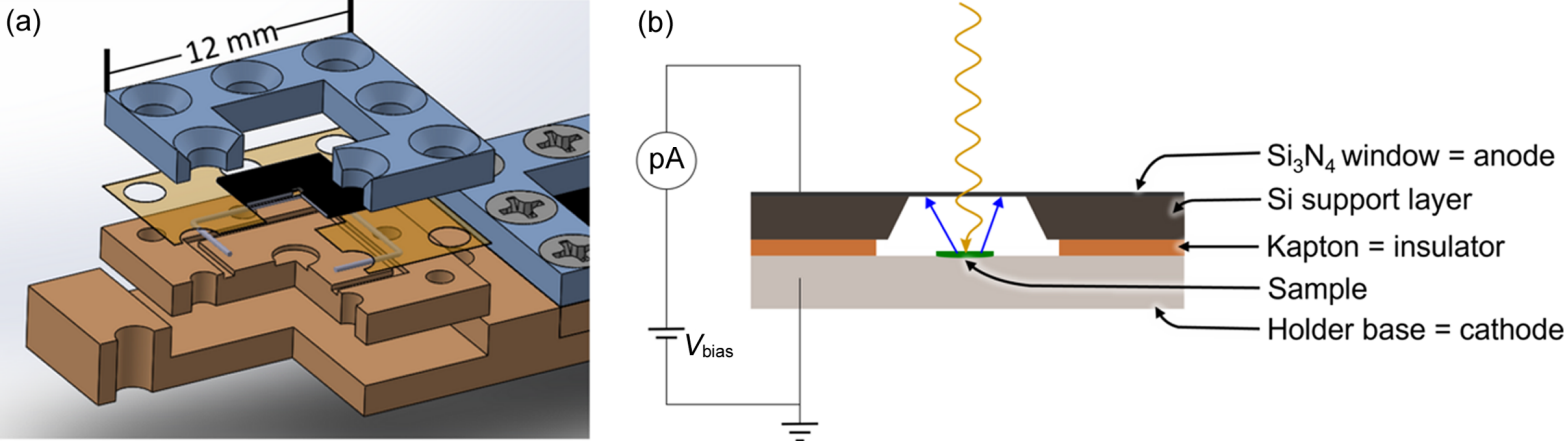
(*a*) Diagram of the holder including the holder base (brown), Kapton gasket (transparent orange), Si_3_N_4_ membrane and Si frame (black), fiberglass (blue), and indium wire (gray). The distance between the sample and the window is less than 0.5 mm (Si support: 200 µm; Kapton gasket: 100 µm; sample well: 100 µm). (*b*) Side-view schematic of the holder with connections and incoming X-rays (yellow arrow) as well as ejected electrons from the sample (blue arrow) during measurements.

The holder base was machined from GlidCop (a metal matrix composite of Cu and Al_2_O_3_) to minimize eddy currents during magnetic spectroscopy while maintaining sufficient thermal conductivity for low-temperature measurements. A small well is machined into the center where the sample can be loaded. The holder may be electroplated with 5 µm Au to remove interference from the Cu *L*-edges (900–1100 eV). Around this well, a channel is milled to accept an indium wire, and at the periphery, 8 holes are drilled and tapped [Fig. 1 ▸, see the supporting information (SI) for a detailed diagram]. The holder base is sealed by an indium wire crush seal, using 8 nonmagnetic stainless steel screws that press a fiberglass ‘frame’ to the base. The total assembled dimensions are 12 mm × 12 mm × 4 mm and the capsule can be sealed in a glove box environment.

The material selection of the X-ray-transparent membrane is critical to the holder design, as it must be robust to breakage and gas diffusion, sufficiently conductive to dissipate charge, and allow soft X-rays to pass through with minimal attenuation. Si_3_N_4_ is mildly conductive, with ρ ≃ 10^10−16^ Ω cm (Vila *et al.*, 2004 ▸; Taft, 1971 ▸) depending on oxygen content, which increases in windows with age (Vogt *et al.*, 2021 ▸). This is most likely an upper bound under experimental conditions, as X-radiation can improve conductivity by several orders of magnitude (Farmer, 1942 ▸; Fowler, 1956 ▸; Weingart *et al.*, 1972 ▸). Coupled with the proven ability of Si_3_N_4_ to contain air-sensitive samples in STXM (Smiles *et al.*, 2020 ▸), we selected these membranes for holder development. The membranes are attached to a Kapton film using thermally conductive but electrically insulating, low outgasing cryo-epoxy (EP21TCHT-1, Masterbond, USA) with a pre-punched hole in the center underneath the membrane. The Kapton is aluminized on the side facing the base to diminish the rate of gas diffusion (Latacz *et al.*, 2023 ▸). This entire window assembly is both air-tight and electrically insulating, and the use of cryo-epoxy enables measurements at liquid He temperatures.

Samples are loaded into the well on the holder base (most samples to date have been prepared by dropcasting). Indium wire is fitted into the machined channel and the window/frame assembly is then placed on top. A protective cover (drawing in the SI) made from polymethyl methacrylate is placed on the assembly to protect the Si_3_N_4_ window during closure. After sealing, the windows are visibly distorted outward by the trapped gas in the sample space; this distortion is robust to vacuum, indicating the integrity of the seal. For work at ALS BL 6.3.1 or 4.0.2, the individual sample holders are affixed to a six-slot adapter. To electrically bias the system, an insulated Cu wire is attached in parallel to the Si_3_N_4_ membranes using conductive silver paint. Typically our approach is to ground the windows and bias the holder base to −10 V or −50 V. This is a diode and detection is achieved by monitoring the current between the sample on the holder base and the window. Charge carriers of this current are electrons emitted in the X-ray absorption process in the sample.

**Figure 2:**
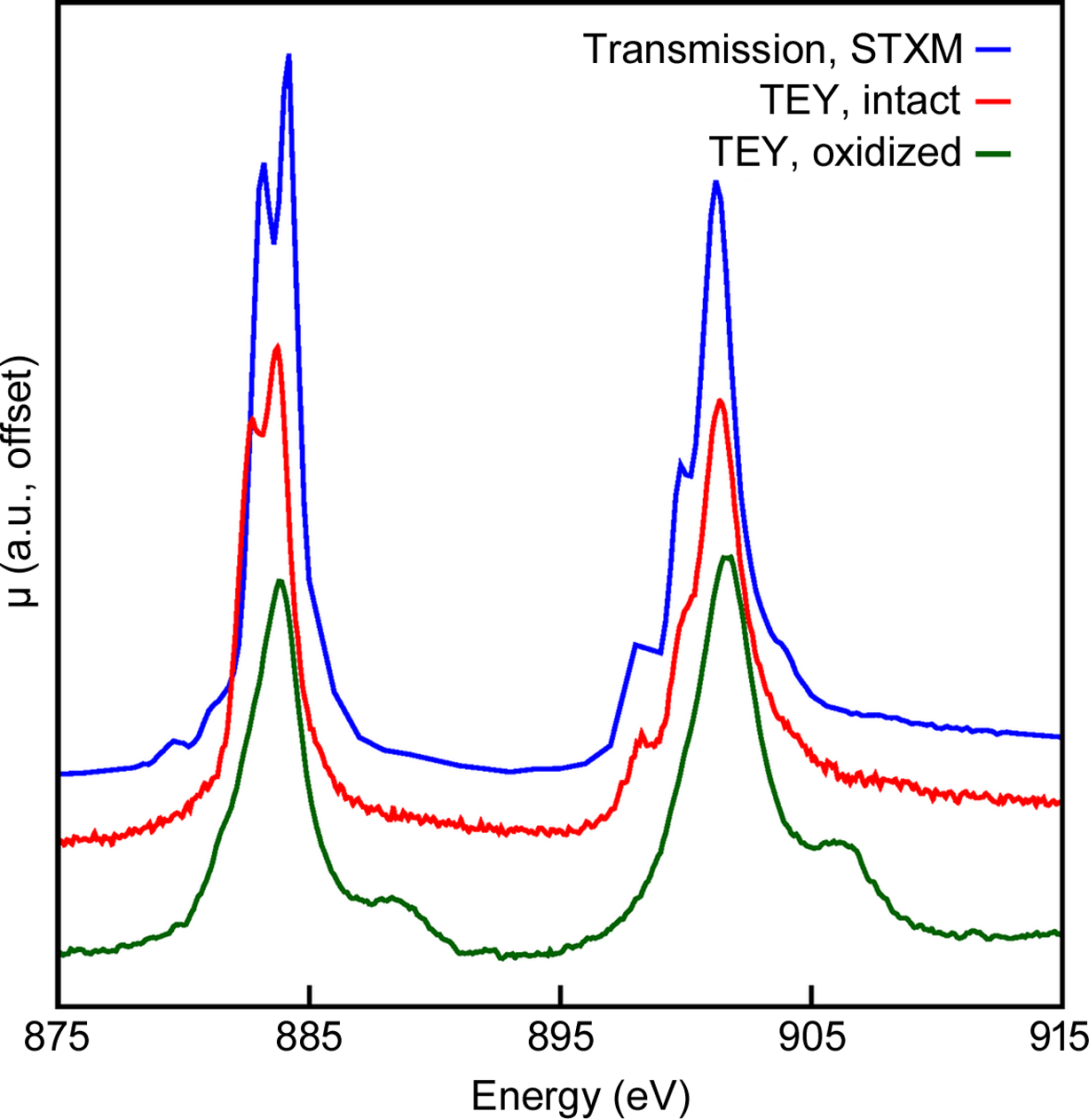
Comparison of *M*_5, 4_-edge XANES of (C_5_HMe_4_)_3_Ce collected using STXM (blue trace) and the current contained EY holder at ALS BL 6.3.1 (red trace, at 77 K). This spectrum may be used to determine sample integrity. Sample oxidation results in the appearance of a new feature at higher energy in both the *M*_5_-edge- and the *M*_4_-edge (green trace).

To prove sample containment, the organocerium complex, (C_5_HMe_4_)_3_Ce, was synthesized and encapsulated in the holder. This highly air-sensitive complex has been used in previous studies (Moreau *et al.*, 2022 ▸) to assess holder integrity, as it is readily oxidized by trace oxygen with accompanying obvious changes both spectroscopically (by the appearance of shoulders in the Ce *M*_5_-edge at 888 eV and the *M*_4_-edge at 908 eV) and visibly (by a change from bright green to nearly black). Trials with a macroscopic amount of material (approx. 5 mg) were performed wherein the sample was loaded into a holder and showed no visible sample degradation after 1 h in air. In a further test, a smaller amount of sample appropriate for spectroscopy was transferred to ALS beamline 6.3.1, where wiring and loading took approx. 30 min. This afforded the spectrum (red trace) in Fig. 2 ▸. This spectrum qualitatively resembles other Ce(III) spectra and is consistent with spectra collected using STXM (blue trace), evidencing no sample degradation (a sample where the seal was intentionally broken is shown in green, Fig. 2 ▸). This result has been reproduced across three separate holders on different days at temperatures ranging from 20 K to 295 K.

## 3. Concluding remarks

There are a number of possible further improvements to this holder that are currently under investigation. Due to their enhanced conductivity, Si or Ge may be a better choice of membrane material than Si_3_N_4_, where higher conductivity may improve electron collection efficiency and consequently signal strength.

The holder described here strikes an effective balance between the conflicting technical needs of maximizing electron yield signal and providing sample containment. Tests showed the holders are spectroscopically robust to at least 30 min handling in air, which is sufficient time for transfer onto beamline holders, wiring and loading into vacuum chambers. The low profile (12 mm × 12 mm × 4 mm) should enable use at many soft X-ray beamlines including those used for XMCD and, with further improvement, may even be used for successive measurements across different beamlines.

## Supplementary Material

Detailed holder drawings and additional information about qualitative testing of the holder. DOI: 10.1107/S1600577524011354/ok5129sup1.pdf
