# Supplementary material for: A fully contained sample holder capable of electron-yield detection at soft X-ray energies
Source: J Synchrotron Radiat. 2025 Jan 1;32(Pt 1):230–3. doi: 10.1107/S1600577524011354 (PMC11708856; doi:10.1107/S1600577524011354)
Supplement: Supplementary file 1 [file s-32-00230-sup1.pdf]

Supporting Information For: A Fully Contained Sample Holder Capable of Electron-Yield Detection at Soft X-ray Energies

S. Olivia Gunther,† Patrick W. Smith,*† Jacob A. Branson, Alexander S. Ditter, Stefan G. Minasian,* Alpha T. N'Diaye,* Bianca Schacherl, David K. Shuh

Lawrence Berkeley National Laboratory, Berkeley, California 94720, United States

† Contributed equally to this work

* email: pwsmith@lbl.gov, sgminasian@lbl.gov, atndiaye@lbl.gov

S1. Supplementary Drawings

=

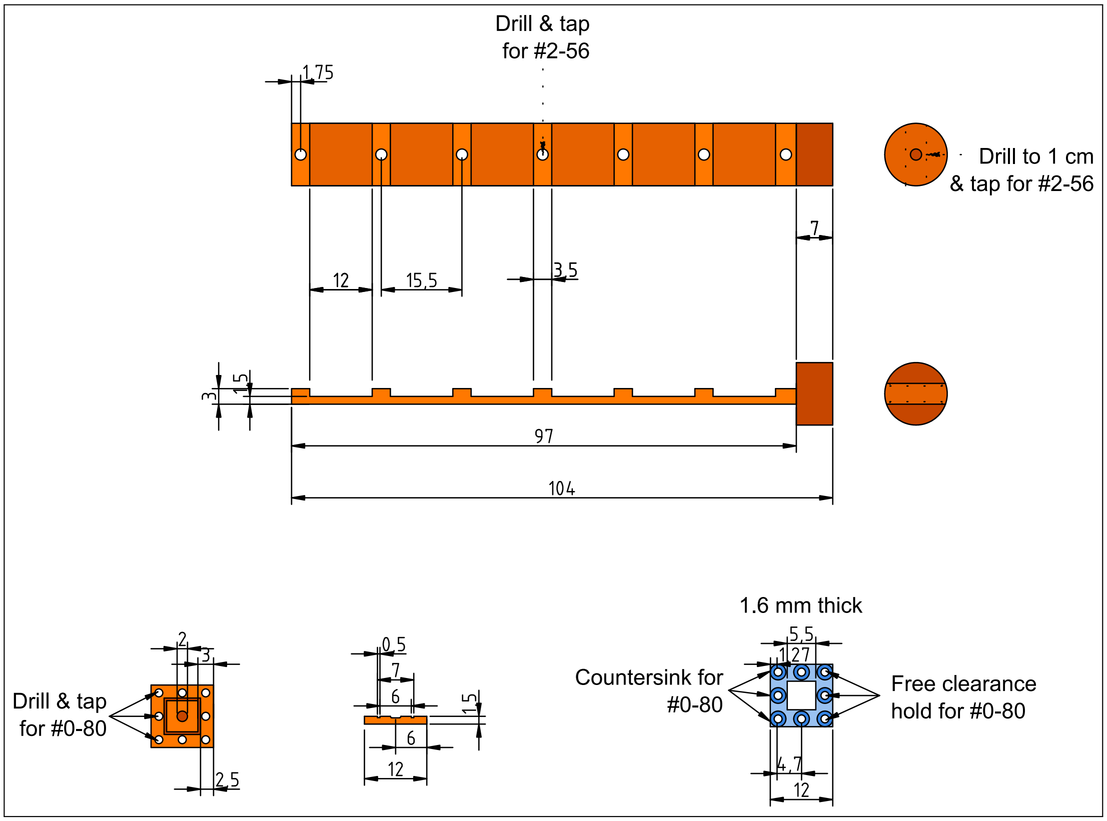

Figure S1. Detailed drawing of the 6-slot adapter used at ALS BL 6.3.1 (top) and the base (bottom left) and frame (bottom right) of the individual holders. Dimensions are in mm. Parts drawn in orange are machined from GlidCop. Parts drawn in blue are machined from fiberglass.

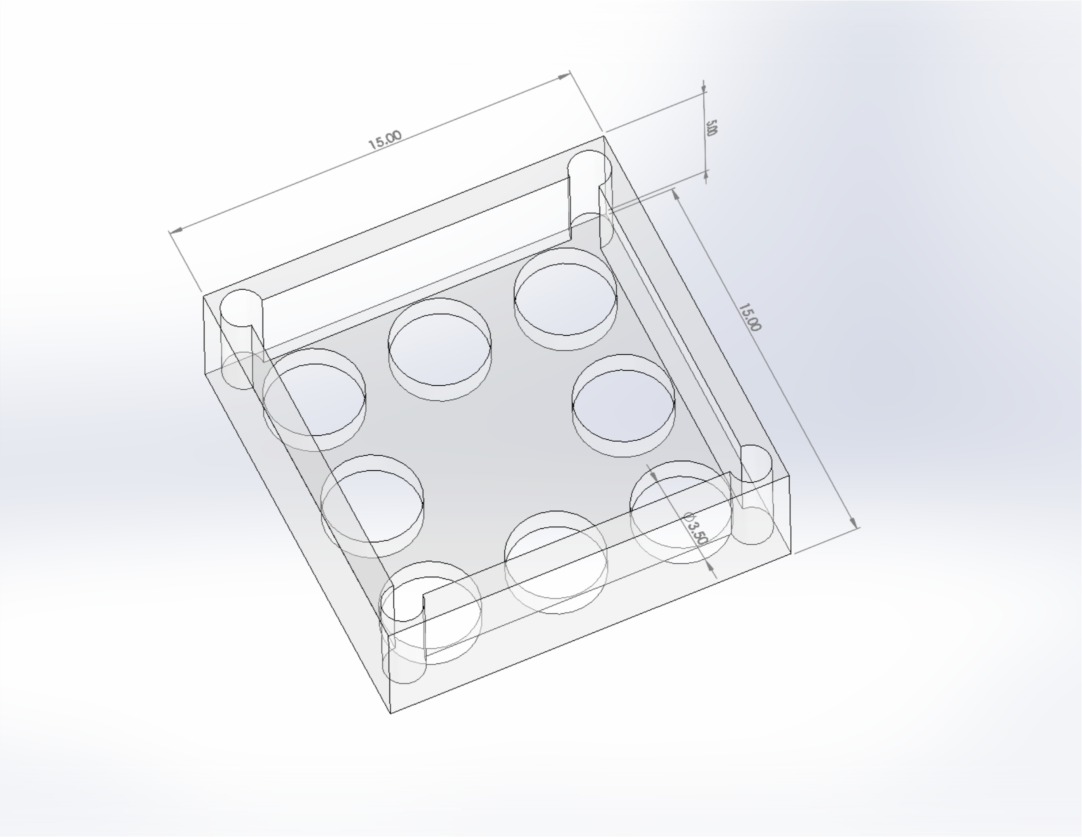

Figure S2. Detailed drawing of the PMMA cover used to protect the Si_3N_4 membrane during holder assembly. Dimensions are in mm.

S2. Qualitative test for the containment of air-sensitive samples

The organometallic complex tris(1,2,4,5-tetramethylcyclopentaienyl) cerium(III) ($(C_5HMe_4)_3Ce$) is green in the 4^+ oxidation state but readily oxidizes in air to a visibly black 4^+ compound. Samples of $(C_5HMe_4)_3Ce$ were loaded into a holder in an argon filled glovebox (Figure S3, left). The holder was affixed with an indium wire, the Kapton-Si_3N_4 window assembly, and crush sealed with the fiberglass frame. A small amount of “puffing” of the Si_3N_4 membrane was observed after assembly, but did not impact the structural integrity of the window. The holder was removed from the glovebox and left on the benchtop in air for >1 hr at ambient pressure. The holder was pumped back into the glovebox after several cycles under vacuum in the small antechamber. After

disassembling the holder in the glovebox, the sample color remained green, suggesting that, qualitatively, bulk samples of $(C_5HMe_4)_3Ce$ survive in the holder assembly long enough to support transportation to the beamline from the glovebox (Figure S3, right). Failed containment tests occurred early in the development process and a visible color change from green to black was easily observed (Figure S4).

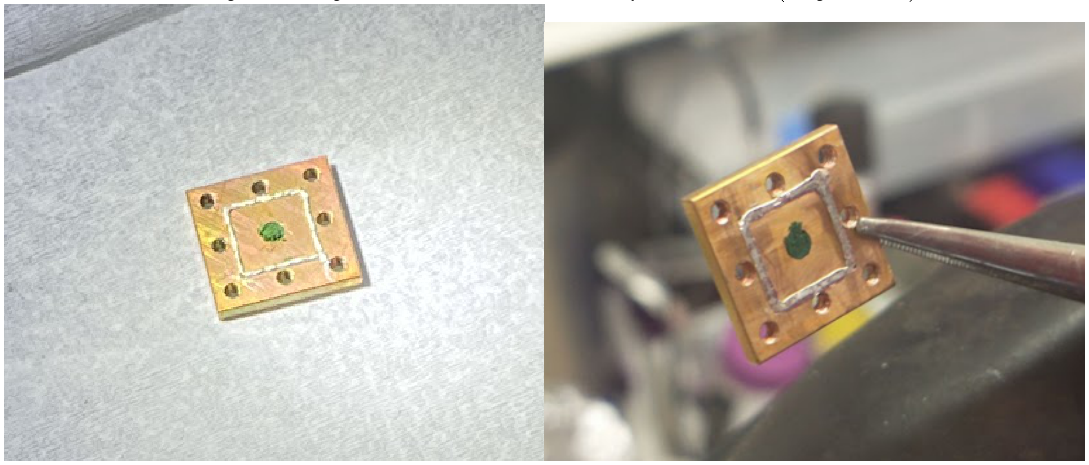

Figure S3. $(C_5HMe_4)_3Ce$ loaded into an XMCD holder base before sealing and exposure to air (left) and upon returning to the glovebox after air and vacuum exposure (right).

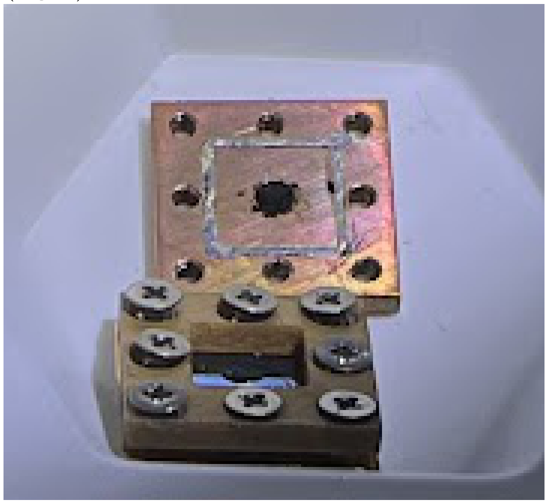

Figure S4. Results of a failed test of containment of $(C_5HMe_4)_3Ce$ after intentional exposure to air. The color of the complex changed from green to black.

S3. Images of the fully-assembled holders

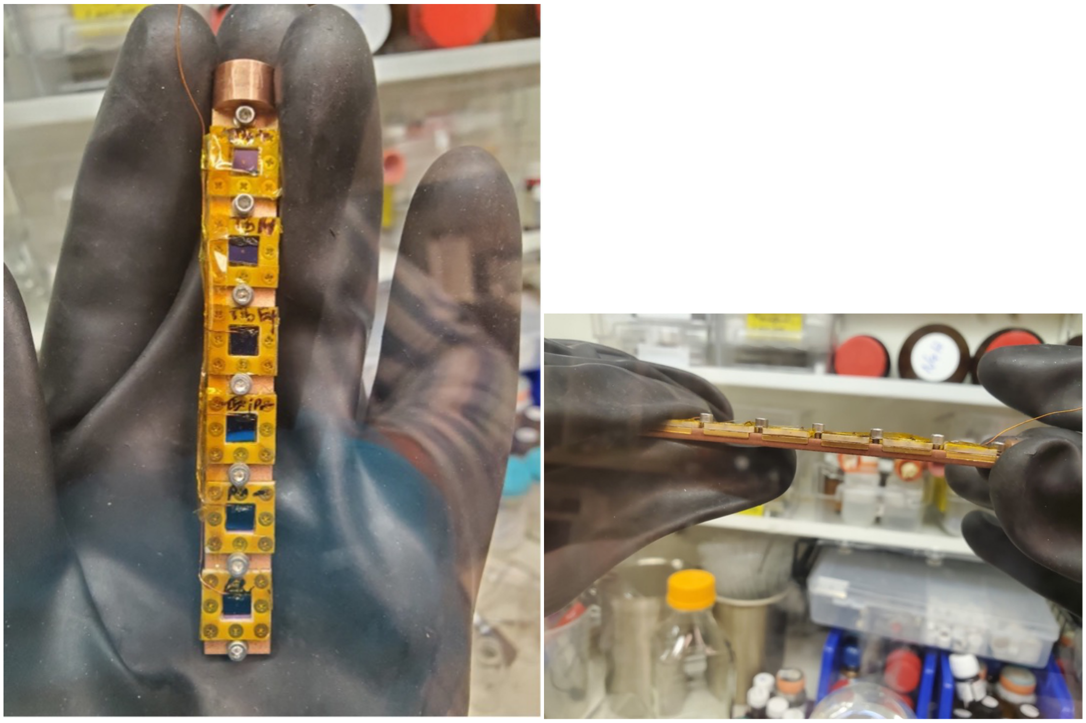

Figure S5. Images of the holder assembly inside of an argon filled glovebox. Before removing from the glovebox, the holder is placed in a 50 mL plastic tube and sealed in two layers of mylar food bags for transportation to the beamline.

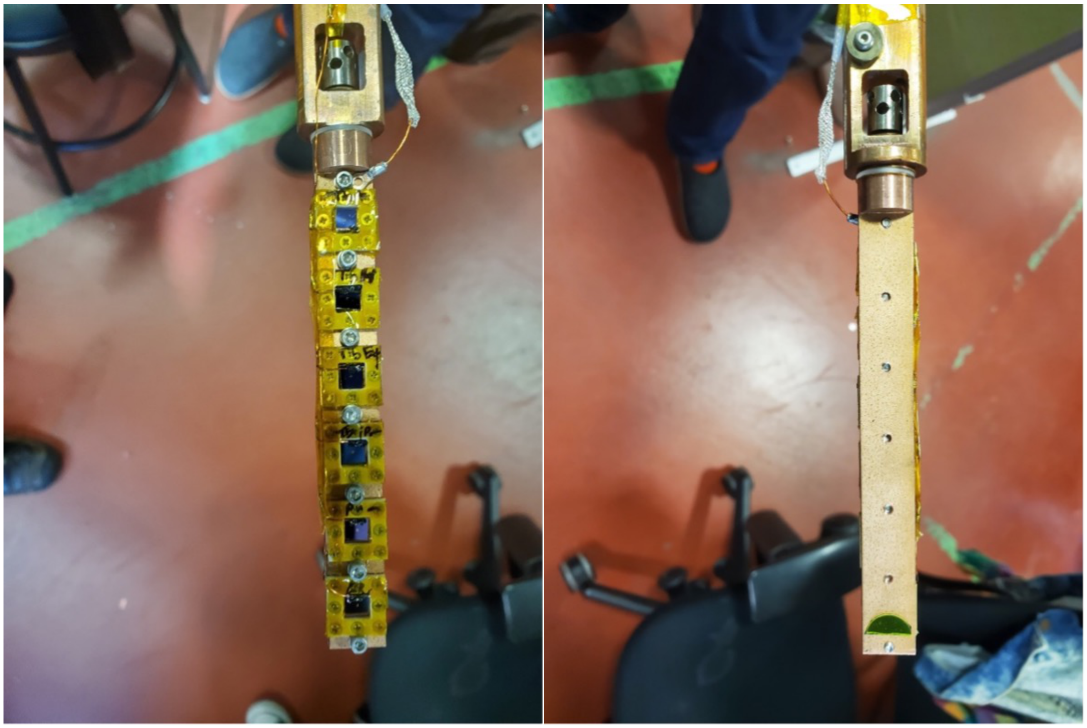

Figure S5. Images of the holder assembly mounted at the beamline.
